# Nature-Based Stress Management Course for Individuals at Risk of Adverse Health Effects from Work-Related Stress—Effects on Stress Related Symptoms, Workability and Sick Leave

**DOI:** 10.3390/ijerph110606586

**Published:** 2014-06-23

**Authors:** Eva Sahlin, Gunnar Ahlborg, Josefa Vega Matuszczyk, Patrik Grahn

**Affiliations:** 1 Department of Work Science, Business Economics and Environmental Psychology, Swedish University of Agricultural Sciences, Box 88, S-230 53 Alnarp, Sweden; E-Mail: patrik.grahn@slu.se; 2 Institute of Stress Medicine, Sweden and Sahlgrenska Academy at the University of Gothenburg, Region Västra Götaland, Carl Skottbergs gata 22B, SE-413 19 Göteborg, Sweden; E-Mail: gunnar.ahlborg@vgregion.se; 3 Department of Social and Behavioral Studies, University West, SE-461 86 Trollhättan, Sweden; E-Mail: josefa.vega.matuszczyk@hv.se

**Keywords:** nature-based therapy, garden activities, sleep quality, burnout, exhaustion disorder

## Abstract

Sick leave due to stress-related disorders is increasing in Sweden after a period of decrease. To avoid that individuals living under heavy stress develop more severe stress-related disorders, different stress management interventions are offered. Self-assessed health, burnout-scores and well-being are commonly used as outcome measures. Few studies have used sick-leave to compare effects of stress interventions. A new approach is to use nature and garden in a multimodal stress management context. This study aimed to explore effects on burnout, work ability, stress-related health symptoms, and sick leave for 33 women participating in a 12-weeks nature based stress management course and to investigate how the nature/garden activities were experienced. A mixed method approach was used. Measures were taken at course start and three follow-ups. Results showed decreased burnout-scores and long-term sick leaves, and increased work ability; furthermore less stress-related symptoms were reported. Tools and strategies to better handle stress were achieved and were widely at use at all follow-ups. The garden and nature content played an important role for stress relief and for tools and strategies to develop. The results from this study points to beneficial effects of using garden activities and natural environments in a stress management intervention.

## 1. Introduction

In the 1990s the Swedish labor market underwent substantial changes, resulting in smaller organizations, staff reductions and reorganizations. This is considered one of the main reasons for the dramatic increase during the early 21st century in sick leave due to stress-related mental disorders such as depression, anxiety, and burnout/exhaustion [1,2].The increase in sick leave due to stress-related disorders at the beginning of the millennium had declined somewhat but is now increasing again, according to a recent report from the Swedish Social Insurance Agency [3]. Before 2011, musculoskeletal diagnoses were the most prevalent reasons for sickness benefit. But since then, mental disorders have become the most frequent diagnoses among women (25% of sick-leave spells) and are predicted to most likely have significant impact on work ability, absenteeism and sick leave for a long time. Adjustment disorder and reaction to severe stress (ICD code F43; about 40%) and depressive episodes (ICD code F32; approximately 30%) are dominant. Exhaustion disorder (ED, ICD-10 code F43) a new diagnosis that has been adopted by the Swedish National Board of Health and Welfare [4], is characterized by mental and physical exhaustion after a long period of obvious stress exposure, accompanied by symptoms such as sleep problems, depressed mood, somatic problems, lack of energy and decrease in cognitive abilities [5]. This leads to a gradual decrease in performance and quality of life, and rehabilitation as well as return to work takes a long time. 

Mental illnesses are also more likely to result in long-term sick-leave compared to other diagnoses and are still, together with musculoskeletal disorders, the diagnoses that most frequently lead to disability pension [6]. The sources of stress are often to be found in increased demands, time pressure and worries connected to work, as well as to stressors in one’s private life, and are of prolonged character [7]. Effects of occupational stress can be absenteeism at the workplace and reduced work performance, connections reported in several studies [6,8,9].

To help keep individuals living under heavy stress from developing more severe stress-related disorders, different stress management interventions are offered by, e.g., occupational health services, public health care, and private health centers. Such interventions can be initiated by the employer, the health care provider or the individual him-/herself.

### 1.1. Stress-related Symptoms

Mental symptoms of burnout, cognitive problems, depression, and anxiety are associated with stress load [5], and somatic symptoms are prevalent effects of prolonged stress. In a meta-analysis of 79 studies [10], the relation between occupational stressors and physical symptoms (gastrointestinal problems, sleep disturbances, dizziness, fatigue, eye strain, headache, appetite and musculoskeletal pain) were investigated. All symptoms were found to have connections to job stressors, gastrointestinal problems and sleep disturbances more than the others. Long-term stress exposure is often associated with lack of recovery and disturbed sleep [11] and, in addition to physical illness, can also lead to mental ill health such as burnout [12], ED [8], depression and/or anxiety [5].

### 1.2. Stress Management Interventions

A great many studies have investigated the effectiveness of different stress-management interventions. Results from a meta-analysis [13] indicate that stress management courses in the surveyed studies were effective, but varied in effectiveness depending on the content of the intervention. The types of intervention studied were: organizational, cognitive-behavioral, relaxation, and multimodal. Results from follow-ups were not included; only pre- and post-intervention results were compared. The outcome variables were grouped in categories relating to: quality of work life; psychological resources; physiology; complaints such as symptoms of stress, burnout, and somatic health; and absenteeism. Only four of the total 48 studies had absenteeism as outcome variable, and none of the intervention types (cognitive-behavioral or relaxation) were successful. According to van der Klink and colleagues [13], the more successful interventions were directed at individuals, while those at organizational level were less beneficial. Since each of the three types of intervention with an individual focus appears to be effective for different types of problems, the authors suggested that it is advisable to choose the type of intervention according to the group’s or individual’s specific needs.Similar results from a meta-analysis based on randomized controlled studies have been reported by Richardson and Rothstein [14], also demonstrating that a shorter intervention had more effect than longer ones. However, this effect was questioned when different intervention types were analyzed separately. Barkham and Shapiro [15] presented positive effects from two short interventions: cognitive behavioral and relationship-oriented treatments, each comprising three therapeutic sessions.

Willert and colleagues [16] reported a significant reduction in self-reported absenteeism from work for participants in a cognitive behavioral stress management program compared to a waitlist control; however, while this difference was in favor of the intervention it had not reached significance at four-month follow-up. A Danish controlled study [17] showed a decrease in self-reported sickness absence for assistant nursing students after a preventive intervention program including stress management, and the intervention group showed significantly less sickness absence than a control group did. However, this positive result did not remain at follow-up 36 months after the program [18] but rather showed increased sickness absence for both the intervention group and the control group.

Werneburg and colleagues [19] reported positive results from a 12-week program, indicating changes to healthier behaviors including physical activities, sleep and eating habits, lowered levels of perceived stress, and improved quality of life, which persisted at one-month follow-up. Results from Internet-based stress management [20,21] indicate that this relatively new model of stress intervention can offer tools for stress management and reduce symptoms of depression and anxiety, but that it also has disadvantages such as high rates of dropout.

In sum, there is some evidence that stress management interventions are effective, although the results reported in the literature are not entirely consistent. It seems as if interventions tailored to the group’s or the individual’s specific needs would be preferable.

A rather new approach is the growing interest in using nature and gardens in stress management contexts. To our knowledge, no scientific studies on the effects of this type of intervention have been published previously.

### 1.3. Nature, Health and Stress

Gardening in therapy has a long tradition. The relation between the environment and human health was described by Hippocrates already in ancient Greece [22]. In the 19th century Benjamin Rush introduced gardening as a therapeutic tool in psychiatry in USA and from there the method spread. During the 1930s horticultural therapy became an established aid to rehabilitation [23,24,25].

Today there is a considerable amount of research exploring different effects on people’s health from being exposed to nature. Earlier studies have shown that these effects relate to a reduction in stress [26,27,28,29,30,31,32,33,34] and an increased cognitive capacity [35,36,37,38,39,40]. Individuals with stress-related mental disorders belong to a vulnerable group which seems specifically to benefit from contact with natural environments. Ottosson and Grahn [41] reported that nature experiences were more valuable in the crisis rehabilitation for individuals in deep crises than those in lower level of crisis. Thorsen Gonzalez and colleagues [42] and Berman and colleagues [40] found beneficial effects of nature interventions on individuals suffering from depression. Johnsen and Rydstedt [43] showed that nature pictures had beneficial effects on emotion regulation, mood, and willpower. Types of natural environment seem to differ in supportive value according to the needs of the individual: Grahn and Stigsdotter [30,31] showed that the higher the perceived stress, the more the individual preferred wild aspects of nature and safe and secluded place (refuge) for recovery. Hartig and colleagues [44] have also discussed the issue from the different, but relevant and interesting, perspective of varying individual needs for psychological restoration related to everyday demands and depleted resources and the possibility of taking advantage of the beneficial health effects associated with exposure to natural environments.

With a background in the research on the relation between health, stress reduction, restoration of depleted resources, and nature, an increasing number of nature-based therapy (NBT) programs addressed to individuals with stress-related illness have started in Sweden in the past ten years. In NBT, traditional methods of treatment and rehabilitation for this group of patients (e.g., pharmacologic, physiotherapeutic and psychotherapeutic interventions, along with occupational therapy and prescribed sickness absence) are used in combination with specially designed activities in specially chosen areas or specially designed gardens to promote recovery [45]. This type of program was originally developed at the Swedish University of Agricultural Sciences [46]. A nature-based stress management course (NBSC) is a new concept, however, and no previous studies on this seem to be available. Since the effects of stress form a major problem in the workplace, for society, and for the individual, it is important that we gain knowledge that can form the basis for developing more effective methods to handle stress and thereby reduce individuals’ risk of developing serious stress-related illness. Thus, this study fills a gap and will provide knowledge about the value of including nature-based methods as a complement to the commonly used stress management techniques.

### 1.4. Aim

The aims of this observational follow-up study were to:

1. Explore whether participation in a nature-based stress management course (NBSC) can influence and change a negative health trend for individuals with increasing stress-related problems.
2. Gain deeper knowledge about how participants experienced and evaluated the nature and garden content in the course.

### 1.5. Research Questions

- Does participation in the NBSC decrease self-assessed burnout and stress-related symptoms as well as sick leave, and increase self-assessed work ability? (Aim 1)
- Do the participants acquire tools and strategies to better handle stress, and are these tools used after the course? (Aims 1 and 2)
- How did participants experience and evaluate the nature and garden content in the course? (Aim 2)

## 2. Method

### 2.1. Recruitment

Region Västra Götaland (VGR), a large public health care organization in western Sweden, is the owner of the course site and also the principal of the course, which is directed exclusively at the region’s own employees in order to prevent sick leave and enhance health. The majority of the employees (approximately 80%) are female. Recruitment to the study was made from six NBSCs held between 2008 and 2010. Information about the course and invitations were e-mailed to HR departments in the health care organization, and were then distributed within the organization to managers and supervisors. Information was also published on the course’s Internet home page. Participants’ admission to the course was preceded by an individual interview held by the psychotherapist.

At the first course assembly, the course leaders distributed a questionnaire constructed for the course evaluation. This first questionnaire was used as the baseline measure in this study. The first author visited each course towards its end and gave information about the study, ethical issues and informed consent, and the opportunity to ask questions was offered. At the last course assembly the team distributed the first follow-up questionnaire to all participants, along with an information sheet and an informed consent form to be signed if the participant agreed to participate in the study. They were also informed that if a participant chose not to sign the informed consent, her questionnaires would only be used for evaluating the course and not for research. As compensation for participation in the study, movie tickets worth 200 SEK (22 EUR, 29 USD) were offered after full participation. The study was approved by the regional ethical board in Göteborg, Sweden (Dnr 472-08).

### 2.2. Subjects and Dropouts

Forty-four individuals started on the six courses, three declined participation in the research and one participant finished after the first course assembly because of illness in the family. Since there was only two male participants in the courses, we decided to include only women in the study. From an initial total of 38 women, three did not return their follow-ups and one cancelled participation because of health problems. One participant were excluded because of severe illness at follow-up. The study participants (*n* = 33) were employed within administration and health care at VGR (for demographic details see Table 1). All participants were invited to an individual interview about their experiences from the course, and 13 signed up for this. Three participants (9%) were on sick leave at course start: one on full-time leave, one 50 % and one 25 %. 

Participants had been notified of the course via the line manager, supervisor or HR department, based on their perception of the participant’s signs of stress and/or repeated short-term sickness absence. Excluded were employees with more than 25% on going sick leave at the time of application for the course and also individuals with walking difficulties. For 22 participants (67%) course participation was suggested by their immediate supervisor, and for three participants (9%) the initiative was taken jointly by the participant and her immediate supervisor. For the remaining 24%, the initiative came exclusively from the participant herself (*n* = 5, 15%) or at the suggestion of a colleague or other person at work (*n* = 3, 9%). 

**Table 1:** Characteristics (count and percentage) of all participants and interviewees.

*Distribution of Participants (Only Women)*	All Participants	Interviewees,
	n = 33	n = 13
	Count (%)	Count (%)
*Age*		
≤49 years	15 (45%)	7 (54%)
≥50 years	18 (55%)	6 (46%)
*Marital status*		
- Married/cohabiting	26 (79%)	9 (69%)
- Single	7 (21%)	4 (31%)
*Educational level*		
- High school	14 (42%)	5 (38%)
- University	19 (58%)	8 (62%)

### 2.3. The Nature-based Stress Management Course

Groups limited to eight participants each met for three hours twice a week for 12 consecutive weeks from March to June or from September to November. Attendance rate for the participants in this study for the 24 sessions was 16–24 (median 23). For the 6 different courses the median for the attendance varied between 20–24. Ninety-one percent of the study participants attended the course 20 sessions or more.

**Figure 1:**
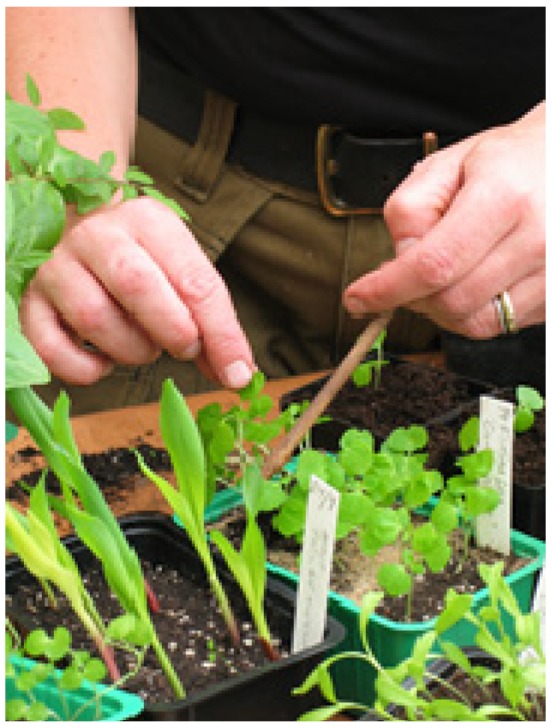
Garden activity (Photo Eva-Lena Larsson).

**Figure 2:**
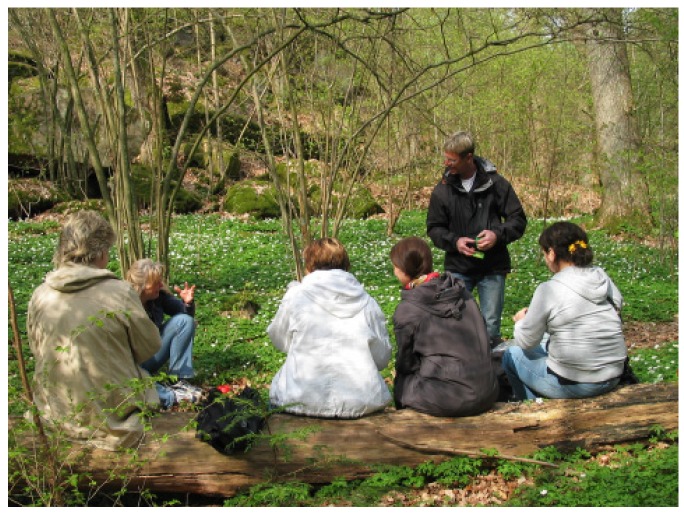
Education about nature during a guided nature walk (Photo Gröna Rehab).

The course was led by a multidisciplinary team composed of a physiotherapist, an occupational therapist, a psycho­therapist (also trained in art therapy), a gardener, and a biologist. The course program included garden activities (Figure 1) following the season, guided walks in the nearby nature reserve (Figure 2), guided relaxation in nature and indoors (mostly mindfulness and breathing techniques), therapeutic painting, therapeutic group conversations, body awareness, and information about stress and stress reactions, a healthy lifestyle, the importance of sleep, and the benefits of physical activities, as well as about nature’s role in health and stress reduction. Garden and nature activities in the course are henceforth referred to as the garden and/ or the nature content.

### 2.4. The Venue

**Figure 3:**
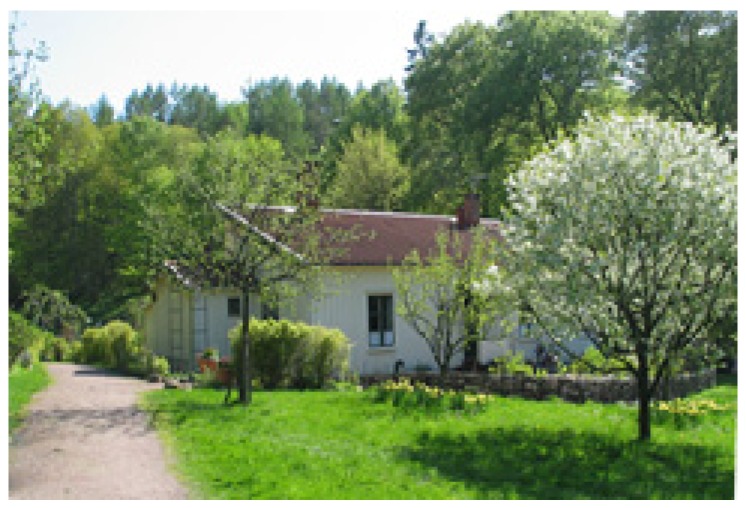
The house with the nature reserve in the background (Photo Eva Lena Larsson).

**Figure 4:**
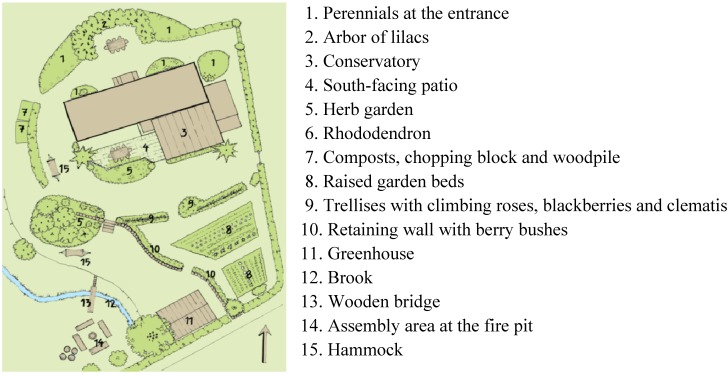
The Venue (Gröna Rehab, Gothenburg Botanical Garden).

The course was held in a small wooden house (Figure 3) with a garden and a greenhouse close to the border of a 225-hectare (555-acre) nature reserve with uncultivated but tended wild nature including forest with deciduous and coniferous trees, moorland, ponds and hills. The site was surrounded by the nature reserve, allotment gardens, and a small brook (Figure 4). The courses were offered during work hours (in the afternoons), at no charge to the participants, and, as customary in Sweden, without reduction in salary.

### 2.5. Measures

**Figure 5:**
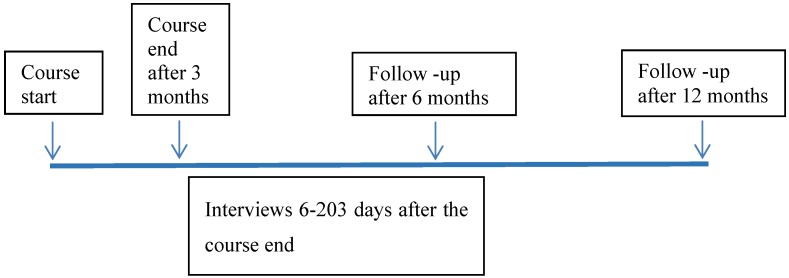
Timeline illustrating the four measuring points: start of course as the baseline measure, the first follow-up at end of course 12 weeks after the course start, and follow-up 6 months and 12 months after end of course and timeframe variation for the interviews.

Measures were collected at baseline (at the end of the first of 24 course sessions) and at the last session (3 months after course start), and 6 and 12 months after the last session follow-up questionnaires were sent by mail to those who had agreed to participate in the study ( See Figure 5).

#### 2.5.1. Quantitative Measures

##### *Primary Measures*:

Burnout: The Shirom-Melamed Burnout Questionnaire (SMBQ) [47]. Since previous results concerning nature’s effects indicate that nature plays a role in restoring mental and emotional exhaustion [48,49] as well as cognitive weariness [39], the SMBQ was chosen as one of two primary measures. This self-assessment questionnaire includes four subscales, two of which are included in Shirom’s definition of burnout [50]: emotional and physical exhaustion (eight items) and cognitive weariness (six items). The other two subscales are tension (four items) and listlessness (four items). In total, the questionnaire comprises 22 items, each with a seven-point response scale (1 = almost never, 7 = almost always). A new validation study has confirmed that the instrument can be used as an overall measure of burnout/stress-related fatigue [51]. The Swedish version of the SMBQ has been used to evaluate treatment effects [52], and correlates highly with Maslach Burnout Inventory [53]. The cut-off used for the SMBQ is often set at 4.0 [5] or 4.6, indicating burnout for scores at or above the cut-off [54]. A cut-off at 3.75 for the SMBQ was chosen for this study, based on the assumption that the subjects had not developed burnout but would still have an increased burnout score compared to an unselected working population, due to their stress exposure [53].

Self-rated work ability: The Work Ability Index (WAI) [55] measures self-assessed work ability. Since effects of stress can lead to reduced work performance [5,6], the development of the participants’ self-assessed work ability during and after the course was assessed using the WAI. The questionnaire includes an assessment of current work ability, diseases, injuries and disorders, absence from work due to illness in the past 12 months, and the person’s perception of his/her health and whether he/she will still be able to be working in the current job two years from now. In this study the first question from the scale was used: *We assume that your work ability, when it is at its best, is valued at ten points. How many points would you give your current work ability? (Please tick the appropriate digit: 0 means you are not able to work, and 10 means your work ability is at its best right now).* The single item from WAI 1 was dichotomized, and new categories were formed by merging the response alternatives 0–8 into one value and the response alternatives 9 and 10 into a second value, based on the assumption that the responses 0–8 were associated with reduced work ability and responses 9 and 10 indicated good work ability [9]. For this study, the question about sick leave (WAI 5) was also used: *How many days during the past 12 months have you been absent from work because of your own illness or injury (care, treatment or examination)?* Response alternatives to this question were: No days, 1–7 days, 8–14 days, 15–24 days, 25–60 days, 61–99 days, and 100–365 days. The response alternatives were dichotomized, with the values *No days, 1–7 days,* and *8–14 days* forming a category indicating low level of sick leave and those *over 14 days* (15–365 days) forming a category indicating high level of sick leave. The reason for this dichotomization is that in Sweden compensation for the first 14 days of sick leave is paid by the employer, while from day 15 compensation is paid by the health insurance. To avoid overlap due to the wide time frame (12-month perspective), only data from course start and 12-month follow-up have been analyzed.

##### *Secondary Measures*:

Based on descriptions in the literature and from clinical experience of stress-related symptoms [5,10] five items were chosen for measuring health symptoms: (1) Stomach pain, nausea, flatulence, loose/hard stools; (2) Pain/ache in the back, neck, joints, knees, *etc.*; (3) Headache; (4) Dizziness; and (5) Heart palpitations, pain/tightness in the chest. There were two possible response alternatives: yes and no. The timeframe for the symptom variables in the baseline questionnaire was the last 12 months, for the questionnaire at the end of the course the timeframe was the last 3 months (during the course), and for the follow- up at 6 and 12 months the timeframe was 6 months.

Since there is evidence that sleep quality is affected by the connection between sleep quality and stress [11], the single-item question from the Karolinska Sleep Questionnaire was chosen for measuring sleep quality: “*How do you find you are sleeping on the whole?*” [56]. The response alternatives were dichotomized, and the values 1 and 2 (*very good; fairly good*) formed a category for reporting good sleep while the values 3, 4 and 5 (*neither good nor bad, quite poor, poor*) formed a category indicating disturbed sleep. The timeframe for this question in all four questionnaires was the same and covered the last 3 months.

#### 2.5.2. Tools and Strategies for Managing Stress

Acquired tools and strategies were measured both quantitatively and qualitatively with questions constructed for the study. Two questions were asked at the end of the course: “*What advice, strategies or tools has the course given you?*” (an open-ended question) and “*After completing the course will you use something of what you have learned?*” (response alternatives: yes, no, I don’t know). These two questions were followed up at 6 and 12 months with: “*What advice, strategies or tools for managing your stress provided by the course do you still use?*”.

#### 2.5.3. Qualitative Measure

Evaluations and experiences of the course were explored through semi-structured interviews following an interview schedule comprised of four themes: (1) Choosing to participate in the stress management course; (2) The work situation; (3) Experiences of the stress management course; and (4) The nature/garden content. The interview started with a question concerning how the participant had heard of the course: “*Who initiated your participation in the course?*”. This was followed by questions about how the course was experienced:“*Describe your experiences of the stress management course*; “*Has the course led to some change in your everyday life? Tell me about it! Can you give an example?*”. Next came questions about the nature/garden content of the course: “*Tell me how you experienced the “green part”* (e.g., the nature and garden content) *in the course*”, including follow-up questions such as “*Has the course given you knowledge about nature/garden that you can use to (better) manage your stress? Can you give an example?*”, and “*Tell me about a situation related to the “green content” of the course that you experienced as important for you and your need to manage stress*”*.* The first three themes were used for interpreting the responses from the questionnaires, and the last theme was analyzed separately to answer to the second aim. All participants were invited to individual interviews, and 13 of the 33 participants accepted and were interviewed after finishing the course. 

### 2.6. Statistical Analysis

Differences in proportions with 95% confidence intervals (CI) between the baseline measures and each of the follow-ups (course end and at 6 and 12 months) were calculated for SMBQ *≤* 3.75, WAI 1 above 8, WAI 5 above 14 days, having good sleep quality, and affirmative answers to the symptom variables. All confidence intervals were calculated according to the method suggested by Newcombe [57]. Although SMBQ items have response scales of ordinal character, mean values with standard deviation are often reported in the literature. Therefore, the SMBQ results in this study will be presented in this customary manner as well as in the way described above.

### 2.7. Qualitative Analyses

The interviews lasted for 26–54 min (total interview time 458 min) and were held in the course house (seven participants), the greenhouse (one participant), at the work place (four participants) or at the first author’s workplace (one participant). All interviews were conducted after the end of the course. The interviewees were all aware of the interviewer’s role as an independent researcher, not involved in the course. Interviews were conducted for nine participants within 3 weeks after course end, with two participants after 47 and 63 days, and two interviews were conducted 176 and 203 days after the end of the course (Figure 1). All interviews were recorded and transcribed verbatim. Each interview was read several times in order to attain thorough understanding. The parts of the interviews involving comments about tools and strategies acquired in the course as well as about the nature and garden content were analyzed in several steps, inspired by the inductive content analysis method [58,59]. The analysis proceeded from writing general comments, summaries and annotations about the content in the margins of the transcript (open coding) to condensing these into shorter meanings without losing the content from the manuscript, and thus creating categories. The categories were listed chronologically in a separate document, and those having connections or showing similarities were grouped under the same headings. The same procedure was followed with all interviews. Lastly, the headings from all interviews were compared to determine whether additional abstraction was possible, and ultimately the themes that had emerged were named. All interviews were analyzed by the first author. To secure trustworthiness, three randomly selected interviews were read and analyzed separately by the first and last authors; the analyses were compared, and no discrepancies were found. For verification each interviewee who was quoted in the article was contacted after the analysis and asked to read and comment, confirm or not agree to the use of the quotations. All confirmed that the quotations and interpretations were correct. 

The procedures in the process followed established principles to ensure highest possible quality [60] and included peer debriefing, participants’ confirmation and procedures for documentation to enable checking and re-checking the data during the entire process.

## 3. Results

At course start, 45% percent of the participants scored *≤* 3.75 on SMBQ and 74% scored 0–8 on WAI 1 (work ability). The relation at baseline between the primary SMBQ and WAI1 measures on the one hand and the other symptoms and sick leave on the other are presented in Table 2. As expected, participants above the SMBQ cut-off and under the WAI1 cut-off, respectively, had more symptoms and long-term sick leave; this was most evident in WAI 1.

### 3.1. Burnout

At course end 48% scored SMBQ ≤3.75, showing only a minor increase compared to baseline. However, greater improvement was seen at 6-month follow-up (73%) and 12-month follow-up (69%); see Table 3. The mean scores decreased at every follow-up (Table 2).

**Table 2:** Distribution at baseline for reporting absence of symptoms and more than 14 days of sick leave for participants scoring *≤* 3.75 and *˃* 3.75 on SMBQ and 0–8 and 9–10 on WAI 1.

Symptoms and Sick Leave	SMBQ		WAI 1	
	*≤* 3.75 15 (45%)	*˃* 3.75 18 (55%)	0–8 23 (74%)	9–10 8 (26%)
Sleep quality	7 (47)	9 (50)	11 (48)	4 (50)
Gastrointestinal symptoms	5 (33)	4 (22)	5 (22)	4 (50)
Pain in the back, neck, knee, *etc.*	5 (33)	6 (33)	7 (30)	3 (38)
Headache	5 (33)	5 (28)	8 (35)	2 (25)
Dizziness	8 (53)	9 (50)	11 (48)	4 (50)
Heart palpitations	7 (47)	9 (50)	10 (44)	6 (75)
Sick leave	6 (40)	10 (56)	15 (65)	0 (n = 7)

**Table 3:** Burnout score on the Shirom-Melamed Burnout Questionnaire (SMBQ) at course start, course end and 6- and 12-month follow-up, presented as count and percentage ≤3.75 and ˃3.75 cut-off and mean and standard deviation (SD).

SMBQ Score	Start	Course End	6-month Follow-up	12-month Follow-up
	n = 33	n = 33	n = 33	n = 32
	n (%)	n (%)	n (%)	n (%)
≤3.75	15 (45%)	16 (48%)	24 (73%)	22 (69%)
˃3.75	18 (55%)	17 (52%)	9 (27%)	10 (31%)
Mean (SD)	3.82 (1.03)	3.56 (1.06)	3.09 (1.21)	2.93 (1.10)

The results in burnout score showed statistically significant differences when comparing scores at start and 6-month and at start and 12-month follow-up, respectively. No significant difference was seen between course start and end (Table 4).

### 3.2. Work Ability and Sick Leave

The results for work ability (WAI 1) indicate a gradual increase in the proportion reporting work ability as good: 9.7% at 6-month follow-up, and 41.9% at 12-month follow-up. The difference between course start and 12-month follow-up was statistically significant (Table 4). The same pattern was seen regarding sick leave (WAI 5), *i.e.*, a clear reduction in the proportion reporting more than 14 days of sick leave during the past 12 months at the last follow-up (Table 4). 

### 3.3. Stress-related Symptoms

In comparison of the absence of stress-related symptoms at start with follow-up at course end as well as at 6 and 12 months, an increase was observed in all of them (Table 4). The largest improvement was in sleep quality (18%), heart palpitations or pain/tightness in the chest, and dizziness (16 and 15%, respectively). The most common symptoms reported at course start were those in the stomach and intestines (73%), headaches (70%), and pain in the back, neck, knee, *etc.* (67%).The smallest improvement (9%) was in pain in the back, neck, knee, *etc.* (Table 5). 

**Table 4:** Differences in paired proportions, with 95% confidence interval (CI) for participants scoring ≤3.75 on the SMBQ, 9 or 10 on WAI 1, and reporting more than 14 days of sick leave on WAI 5, respectively. Comparing start of course with follow-up at course end, and 6 and 12 months, respectively.

Length of Follow-up	Start	Follow-up	Difference	95 % CI
	*%*	*%*		
SMBQ ^1^				
End of course (n = 33)	45.5	48.5	3.0	−13, 18.8
6 months (n = 33)	45.5	72.7	27.3	10.4, 41.6
12 months (n = 32)	46.9	68.8	21.9	3.7, 37.7
WAI 1 ^2^				
End of course (n = 31)	25.8	32.3	6.5	−12, 24.5
6 months (n = 31)	25.8	35.5	9.7	−9.9, 28.3
12 months (n = 31)	25.8	51.6	25.8	6.3, 42.5
WAI 5 ^3^				
End of course (n = 31)	51.6	41.9	−9.7	−20.8, 2.2
6 months (n = 30)	50.0	40.0	−10.0	−26.6, 7.7
12 months (n = 31)	51.6	9.7	−41.9	−59.5, −19.3

Notes: **^1^** The Shirom-Melamed Burnout Questionnaire: assesses emotional and physical exhaustion, cognitive weariness, listlessness and tension. **^2^** WAI 1: single-item question on self-assessed work ability. **^3^** WAI 5: single-item question on sick leave during the past 12 months.

**Table 5:** Stress-related symptoms (count and percentage) reported by participants at course start, course end and 6- and 12-month follow-up.

Stress-related Symptoms	Course Start Count (%)	Course End Count (%)	6-month Follow-up Count (%)	12-month Follow-up Count (%)
Self-rated sleep quality				
Good sleep	16 (48)	16 (48)	20 (61)	22 (67)
Disturbed sleep	17 (52)	17 (52)	13 (39)	11 (33)
Symptoms in the stomach and intestines				
yes	24 (73)	17 (52)	19 (58)	20 (61)
no	9 (27)	16 (52)	14 (42)	13 (39)
Pain in the back, neck, knee, *etc.*				
yes	22 (67)	22 (67)	20 (61)	19 (58)
no	11 (33)	11 (33)	13 (39)	14 (42)
Headache				
yes	23 (70)	22 (67)	22 (67)	19 (58)
no	10 (30)	11 (33)	11 (33)	14 (42)
Dizziness				
yes	16 (48)	10 (30)	9 (27)	11 (33)
no	17 (52)	23 (70)	24 (73)	22 (67)
Heart palpitations, pain/ tightness in the chest				
yes	17 (52)	9 (27)	11 (33)	12 (36)
no	16 (48)	24 (73)	22 (67)	21 (64)

The proportion of participants reporting *good sleep* increased between start and 6-month follow-up, and even more between start and 12-month follow-up; however, it did not reach formal statistical significance (Table 6). Significant changes in *gastrointestinal symptoms* were seen between course start and end, as indicated by the confidence interval (1.6, 38.5) showing an increase in the number of participants reporting no problems from the stomach and intestines. Although there was an increase (Table 5) in the number of participants reporting no gastrointestinal symptoms at 6- and 12-month follow-up compared to course start, the significant change was not sustained (CI −3, 31.9 and −7.1, 30.1); see Table 6.

The differences between start and the follow-ups were too small (Table 5) for *pain in the back, neck, knee, etc.* and *headache* to show any significant differences. Concerning *dizziness*, significant change was seen between course start and end (CI 1.4, 33.3), with fewer participants reporting this symptom. There were positive changes between start and 6-month follow-up (21 percentage points), with fewer participants reporting dizziness, but this did not reach formal statistical significance (Table 6).

**Table 6:** Differences in paired proportions with 95% confidence interval (CI) for participants reporting absence of the respective stress-related symptoms. Comparing start of course with follow-up at course end as well as at 6- and 12-month follow-up, respectively (n = 33).

Length of Follow-up	Start	Follow-up	Difference	95% CI
	*%*	*%*		
*Sleep Quality*				
End of course	48.5	48.5	0.0	−16.9, 16.9
6 months	48.5	60.6	12.1	−8.6, 31.3
12 months	48.5	66.7	18.2	−0.6, 35
*Gastrointestinal Symptoms*				
End of course	27.3	48.5	21.2	1.6, 38.5
6 months	27.3	42.4	15.2	−3.0, 31.9
12 months	27.3	39.4	12.1	−7.1, 0.3
*Pain in the Back, Neck, Knee, etc.*				
End of course	33.3	33.3	0.0	−19.0, 19.0
6 months	33.3	39.4	6.1	−13.0, 24.5
12 months	33.3	42.4	9.1	−9.1, 26.3
*Headache*				
End of course	30.3	33.3	3.0	−11.3, 17.2
6 months	30.3	33.3	3.0	−11.3, 17.2
12 months	30.3	42.4	12.1	−2.9, 26.3
*Dizziness*				
End of course	51.5	69.7	18.2	1.4, 33.3
6 months	51.5	72.7	21.2	−0.2, 40.0
12 months	51.5	66.7	15.2	−6.4, 34.7
*Heart Palpitations, Pain/Tightness in the Chest*				
End of course	48.5	72.7	24.2	3.9, 41.7
6 months	48.5	66.7	18.2	1.4, 33.2
12 months	48.5	63.6	15.2	−0.8, 29.6

A significant difference was seen for *heart palpitations, pain/tightness in the chest*, with fewer participants reporting this symptom between course start and end, and between start and 6-month follow-up. There was still a difference between start and 12-month follow-up, but it did not reach formal statistical significance. No other significant changes concerning health symptoms were seen.

### 3.4. Use of New Tools and Strategies for Managing Stress

At course end, all participants reported that they had acquired tools and strategies during the course, and the majority reported continued use of these at the follow-ups. A minority of the participants did not use any tools or strategies from the course (Table 7). The most frequent responses to the question “*What advice, strategies or tools acquired from the course for managing your stress are you still using after the course?*” were, firstly, using relaxation and breathing techniques and, secondly, taking walks in nature and observing and enjoying nature more while walking; just as often, they mentioned limiting engagement, “saying no”, and taking breaks (Table 7). Comments were also made about being more physically active for fitness (such as taking more walks, biking to work, exercising regularly) and engaging in creative activities for pleasure and joy (results not shown).

**Table 7:** Participants’ use of new tools and strategies for stress management at course end and at the 6- and 12-month follow-up. An unlimited number of responses was possible for this open-ended question.

Use of New Tools and Strategies n = 33	Course End Count (%)	6 Months Count (%)	12 Months Count (%)	A Selection of Responses from Participants at 12-month Follow-up regarding How Tools/Strategies Help in Stress Management.
*Use of new tools and strategies*				
yes no	33 (100) 0	31 (94) 2 (6)	31 (94) 2 (6)	
*Relaxation/Breathing techniques*	23 (67)	28 (85)	26 (79)	*“I can detect early on when stress takes over—and then withdraw for breathing and mindfulness.”* *“Focus on breathing and body awareness.”*
*Using gardening/Nature to handle stress*	16 (48)	8 (24)	8 (24)	*“Nature walks during leisure”*. *“When stressed, I watch trees and how their leaves are gently blowing.”* *“With eyes open, see the small things and details in nature.”*
*Say “no”;* *limiting engagement;* *taking breaks*	16 (48)	16 (48)	20 (61)	*“Taking small breaks, daring to say no, letting go of the need to control.”* *“Listen to signals from my body.”*

### 3.5. Qualitative Results

Three main themes emerged: Education about nature and garden; The impact of the environment; and Tools and strategies for managing stress.

#### 3.5.1. Education about Nature and Garden

Guided walks in the nearby nature reserve were included in the course. This “education” about nature’s details and processes awoke “*curiosity I haven’t experienced before*” and opened up to the beauty and ingenious mechanisms of nature. The experiences in nature also opened up for existential reflections to emerge: “*Now, let me say that I’m a deeply devoted atheist so I do not believe in any Creator, but nonetheless I can almost have one of those religious experiences from being in nature, think it’s fantastic. And they’ve indeed conveyed that very well... That you’re part of a context—making you new*”*.*

Hence, nature was experienced with greater enjoyment and made participants value their own nature walks more than before the course: “*If I walk in the forest now, I see, I look at other things now and in a way I’ve never done before*”*.*

The new knowledge about nature became a topic for conversations in different contexts; in quizzes and social chatter at coffee breaks, and also in educating family or friends: “*So if we had nothing to do or when there was a nice weekend or something, the whole family went there and I guided them in the same way as I’d heard it from the team*”*.*

However, some participants found it difficult to find time after the course to take these nature walks they knew would provide relaxation and stress reduction, saying “*I’ll do it later*” or “*I’ll do it when spring comes…*”*.*

The joy and usefulness in learning more about gardening and plants were described as tools; not only in terms of coping with stress but also for personal well-being. In the garden activities, the participants became focused and could concentrate. Many participants have their own garden or balcony, and several described that after the course they had greater pleasure during the growing season: “*I’ve had a garden for 40 years and I have never felt this way. Before it was more about tidying up in the garden*”*.*

#### 3.5.2. The Impact of the Environment

Participants expressed that they experienced tranquility, stress relief and peacefulness when in nature or in a garden. “*This thing with the forest and gardens is really the perfect way to relax*”. One participant made a clear distinction between the value of a garden and that of nature experiences:“*In the garden you’re more active yourself, you’re doing something in the garden; while in nature you just absorb the beauty, in a different way*”*.*

Several emphasized that they normally worked during the day, and that participating in the course gave them the possibility to “*refuel with daylight*”*.* Entering the course site (or approaching the house) was experienced as entering “another world”, away from work and everyday chores, offering stress relief: “*already when crossing the bridge* (approaching the house) *I felt the stress folding away*”, or as described by another participant, “*because you feel so good when coming up to this place and you just notice* (exhaling audibly with delight) *the serenity—then it’s like you don’t care about that traffic noise* (far away) *even though it can be heard*”*.*

#### 3.5.3. Tools and Strategies for Managing Stress

The tools and strategies for managing stress acquired in the course were described by the majority as being used after the course; however a few participants declared that, against their better judgment, they did not use the tools from the course to the extent they knew would benefit them.

The relaxation techniques of breathing and mindfulness were mentioned most frequently as valuable tools, and were of great help in handling stressful situations: “*Yes I’ve gotten a tool that I’ve incorporated: to inhale the fresh and new and to exhale and let go of the old, the wasted. That suited me so well*”*.*

Other tools and strategies mentioned were the ability limit engagement, “*to concentrate on one thing at a time*”, and “*actually say no and take a break without having to feel ashamed*” when too much pressure was experienced in the work situation. Another strategy was the ability to more consciously and quickly recognize one’s own signs of stress and use tools acquired during the course in these situations to dissolve the escalating stress: “*Although several weeks have passed* (since the course) *it’s still left there as a remembrance in my body, and when I need to relax I just take these two breaths…and it* (the stress) *disappears*”*.*

For several participants work in the home garden had become more pleasurable and undemanding, which was directly derived from garden activities in the course and education about gardening and plants. Hence, they spent more time in their own garden for well-being and being more physically active, but also for stress relief: “*Before* (the course) *it was a bit tough and stressful to deal with weeds, but now it’s almost meditation when I’m out pulling the weeds one by one; yes, I don’t feel so stressed out by it anymore. It takes the time it takes, and that feels almost relaxing*”*.* After the course, nature walks were used more frequently as a tool for stress relief, to restrict tiring and demanding stimuli, and for exercise. Mental images had been “stored in the mind” from the time spent in the course, and these images could be picked up in stressful situations and thus bring the participant mentally back to a positive situation they had experienced during the course, hence giving them a relaxing effect or a reminder to act according to strategies practiced during the course: “*I’ve evoked images* (from nature experiences in the course) *several times, images when I’m in some stressful situation* (at work)…*images of how to relax, as well as taking two deep breaths*”*.* Objects or work taken home from handicraft or gardening activities gave similar effects of serving as reminders.A few participants described the outdoor guided relaxation as sensory-intrusive and thereby disturbing the ability to relax: “*There were this buzzing bumblebee and the grass tickled when we lay down. And the sun came up and warmed the air, and the birds were twittering. It was too much! Too many impressions!*”. However, an overwhelming majority described the relaxation sessions outdoors in nature as superior to the relaxation indoors, with the narratives stating that deeper sensory experiences (“*The birds singing, you feel the sun, the wind - the senses become somehow more alert...*”) during the outdoor guided relaxations contributed to a deeper relaxation and a better mood: “*It may have been the sounds; there was a light breeze, and it made you a bit happy to hear the birds*”*.*

## 4. Discussion

The main results from the study were that the level of self-assessed burnout had decreased, work ability had increased, and long-term sick leave had decreased at the follow-ups. Burnout symptoms declined more rapidly, while experienced work ability had clearly increased only at 12-month follow-up. All health symptoms showed improvement; *i.e.*, a larger proportion of participants reported absence of symptoms. However, it was only in some comparisons that significant differences were reached. The participants acquired tools and strategies to better manage their stress, which was confirmed in both the qualitative and quantitative analyses. Additionally, the nature and garden content was of importance for stress relief and for these tools and strategies to be developed, as well as for the continued use of nature and garden activities for stress relief after completing the course.

### 4.1. Burnout, Work Ability, and Sick Leave

The positive effects found for burnout and work ability may partly be explained by the use of tools and strategies for managing stress acquired through the course, but also by the participants’ continued use of nature for stress recovery, sensory experiences and physical activities after the course, which a large body of earlier research has shown [28,30,31,33,61,62] to have beneficial effects on stress and well-being. More frequent walks in nature, work in one’s own garden, and more regularly practiced physical activities after the course were reported in the questionnaires used in the study as well as in the interviews, and may have contributed to the increase in work ability. A positive relation between work ability and physical activities has been shown by Arvidsson *et al.* [61].

The proportion of participants who rated their work ability 9-10 doubled between course start and 12-month follow-up**.** One plausible explanation for seeing these changes from the longer perspective may be that it takes time to change dysfunctional patterns of behavior and reaction, and develop and manifest new (healthier) habits and experience positive consequences from changes in one’s approach to stress. Hence, the non-significant result between course start and end/six-month follow-up may not be surprising. 

Moreover, the results showed a large and impressive reduction in long-term sick leave, in the proportion 5:1 between course start and 12-month follow-up. Few studies have used sick leave as an outcome measure as was done in this study, thus making it particularly interesting. Van der Klink and colleagues [13] found no study reporting a positive effect on sick leave. Contrary to Willert *et al.*’s [16] results for effects on sick leave in the longer run, our results showed a positive development, indicating a beneficial long-term effect of the nature-based stress management course. The reduction in longer sick leave in favor of shorter leave at 12-month follow-up may, however, disguise a coping behavior, as suggested by Kristensen [63]. As shown in the results, after the course the participants were more responsive to their own needs; thus, short-term absences may have been used to reduce work strain and offer recovery, but what speaks against this interpretation is that nothing attesting to such reasoning emerged in the interviews. 

### 4.2. Health Symptoms and Sleep Quality

Gastrointestinal symptoms and sleep quality are often adversely affected by stress problems, as described in the introduction to this study [10]. A majority of the course participants reported gastrointestinal symptoms at course start, and positive significant changes were seen between course start and the follow-up at course end, but leveled off at 6 and 12 months. At the baseline measure almost half of the study population reported impaired sleep. Sleep disturbances have been reported to predict future long-term sickness absence, and may play a role in the development of fatigue and burnout [64,65]. The proportion of participants reporting having good sleep had increased at 6- and 12-month follow-up, but did not reach significance (however it came very close at 12 months). The results in this study indicate better sleep quality in the longer perspective, which may be explained partly by participants using relevant tools from the course such as relaxation, walks in nature and being more physically active, which have been shown to promote sleep. Also, being outdoors during daylight may play a role, as daylight is vital to our circadian rhythm and in regulating sleep and alertness. Nature-based therapy and nature contacts have been shown to significantly reduce stress symptoms [27,31,32,66]. A possible explanation for this could be that physical stress arousal has been shown to be more easily and quickly normalized in contact with nature environments with restorative qualities [26,27,67]. It was promising to see that long-term effects were demonstrated in the results; although the study of course cannot answer whether these changes would have appeared anyway. However, the inclusion criterion for the course was the existence of accelerating stress symptoms over a longer time period, and it could be assumed that an intervention was probably necessary to alter this development. A well designed controlled study is needed for this to be thoroughly explored.

### 4.3. Tools and Strategies for Managing Stress

The use of acquired tools for managing stress was at almost identical levels at both 6- and 12-month follow-up, indicating that they were more permanent, which was also obvious in the qualitative part of the study. The very few participants reporting that they did not use any tools or strategies at the follow-ups may have integrated them so firmly into their behavioral repertoire that they no longer considered them new; however, this is an interpretation that cannot be confirmed in this study.

Activities in nature and one’s own garden were perceived after the course as more fun and rewarding. These results are in line with what was found in the theme “changing dysfunctional patterns of thoughts/behaviors” by Sahlin *et al.* [62], and also with Palsdottir *et al.*’s [66] results on the importance of experiencing feelings of self-reward in connection to nature and activities in nature. The results clearly showed that the participants were more responsive to their own needs, as shown for instance in their engaging in crafts and creative activities that affirmed their own value, as well as their acknowledgment of the need to indulge in “me time” to replenish their energy and power. It has been reported in a Norwegian study [43] that exposure to nature pictures had some beneficial effects on mood and emotion regulation and also that expecting such beneficial effects may contribute to individual’s inclination to seek nature environments. According to these findings it may be possible that the more frequent nature contacts that were reported in our study after the course, and also more pleasurable engagement in gardening, may be explained by the enjoyable and rewarding experiences of nature and garden during the course followed by expectations of beneficial effects when taking a walk or when working in the garden after completion of the course [43].

### 4.4. The Nature and Garden Content

The participants expressed satisfaction with the course, and stated that they had experienced the joy of activities at a moderate tempo and in an undemanding environment corresponding to their needs. Some underlined the importance of spending time outdoors, which besides the contact with nature also offered the positive effects of daylight exposure. However, most prominent was the participants’ experience of the guided nature walks, which encouraged them to start up new hobbies or resume old ones, such as photography, but which also offered them new experiences and knowledge about nature and inspired them to take more walks on their own after completing the course. This knowledge about nature gave them a new perspective on its value, and contributed to more profound enjoyment of experiencing it in different social contexts.

The nature environment gave a feeling of being away from the everyday stress, which is in line with Attention Restoration Theory (ART) by Kaplan [35]. According to ART, depleted directed attention can be restored in a restorative environment containing qualities of “being away” (*i.e.*, from the everyday situation), “compatibility” (meeting the needs of the individual) and “soft fascination” (soft sensory impressions that do not intrude, such as rustling from the trees, birdsong, scents of nature). All these qualities are expressed in the participants’ narratives. Kaplan [48] also suggests that education (about nature and its benefits for health and well-being) and meditation may enhance the restorative effects in a restorative environment, which seems to be the case for this study population. This may explain the descriptions in this study about achieving deeper relaxation when performed in nature compared to indoors.

For clinically exhausted individuals, nature experiences during a nature based therapy program was shown to mediate existential reflections to start [62] which were experienced as vital in the rehabilitation process. Similar, but less profound, reflections were found in the analysis of the interviews in this study, indicating that experiences of nature may be of importance for recovery when affected from stress at different levels of severity. 

### 4.5. Additional Reflections

The research question for the qualitative part of the study focused on the value of the garden/nature content (the part of the intervention that focused on activities in garden and/or nature) the program which has been discussed in the section above. But also the social environment, including the team and other participants, was of importance. There were statements on the team’s beneficial impact in acquiring tools and strategies for stress management and the importance of the other participants in the course was also obvious. To be among individuals with similar difficulties and/or symptoms was regarded as helpful. This had also been reported earlier by other authors, for instance Thorsen Gonzalez *et al.* [42] Sahlin *et al.* [62], and in Palsottir [68].

### 4.6. Strengths and Limitations

Qualitative as well as quantitative approaches have been used here; thus, the research questions have been highlighted from different perspectives and the results from the questionnaires have been elucidated from the interviews. Self-assessment questionnaires are the commonly used measures for health, as well as for experienced burnout and work ability. A strength of this study is that the results are based on validated instruments or validated parts of instruments; furthermore, the 12-month follow-up is a longer timeframe than several other studies in this field have used to follow up effects. However, we have relied on self-reported sick leave from a long perspective; 12 months may be a long time frame for remembering, and therefore there is a risk that data are not as correct as they would have been if we had used absence data from the employer or the Social Insurance Agency. The CIs are wide and the estimates are thus uncertain, as a result of the small study population. 

We have illuminated experiences and results from six different courses during different seasons, which gives a broad base for summarizing the qualities and benefits of this specific nature-based stress management course; however, we have not been able to explore any gender-specific results as there were no male participants included in the study. Even though the study population includes participants from six different courses, this was still not enough to conduct further analysis in subgroups. These questions are suggested topics for further research together with whether there may be seasonal differences due to beneficial effects from sunlight on, for instance sleep, quality.

One of the reasons for attending the course was stress-related problems along with repeated absence, but we have not asked the reasons for the sickness absence either at baseline or 12 months. However, we see no way that sick leave change before course start to12-month follow-up could be explained by other causes of disease than those associated with stress.

Since this study was to our knowledge the first to follow up the effects of a stress management course including the use of nature and garden it had an explorative design; *i.e.*, no control or reference group was used. It is desirable that future studies use a controlled design, comparing a “green stress management” intervention with a standard stress management one without nature/garden content.

## 5. Conclusions

The levels of stress measured through self-rated burnout, somatic symptoms and sleep quality declined, and continued to decline to the last follow-up. Along with this improvement in health, the participants’ self-rated work ability increased and a great change from long to short sick leave was observed. The qualitative part of the study demonstrates the profound appreciation of the nature and garden content of the course. The participants perceived that the environment in itself had a relaxing effect, and there was a clear indication that these experiences provided them with nature-based tools and strategies for stress management, which they continued to use for stress relief.

The education during the guided walks can also be said to have influenced the participants to take nature walks more frequently after the course end, thus contributing to a healthier lifestyle. 

The participants acquired tools and strategies to better handle stress. In addition to what is obtained from ordinary stress management courses—e.g., listening to one’s own needs and bodily signals, limiting engagement and using breathing techniques and relaxation in stressful situations and for insomnia—they stored mental images from joyful or peaceful situations during the course, which could be evoked in problematic situations and thus serve as a tool for reducing feelings of stress. The results from this study point to good effects from using garden activities and natural environments in a stress management context.
